# Dissociable Effects of 5-HT2C Receptor Antagonism and Genetic Inactivation on Perseverance and Learned Non-Reward in an Egocentric Spatial Reversal Task

**DOI:** 10.1371/journal.pone.0077762

**Published:** 2013-10-30

**Authors:** Simon R. O. Nilsson, Elizabeth M. Somerville, Peter G. Clifton

**Affiliations:** 1 School of Psychology, University of Sussex, Brighton, East Sussex, United Kingdom; 2 School of Life Sciences, University of Sussex, Brighton, East Sussex, United Kingdom; University of Iowa, United States of America

## Abstract

Cognitive flexibility can be assessed in reversal learning tests, which are sensitive to modulation of 5-HT_2C_ receptor (5-HT_2C_R) function. Successful performance in these tests depends on at least two dissociable cognitive mechanisms which may separately dissipate associations of previous positive and negative valence. The first is opposed by *perseverance* and the second by *learned non-reward*. The current experiments explored the effect of reducing function of the 5-HT_2C_R on the cognitive mechanisms underlying egocentric reversal learning in the mouse. Experiment 1 used the 5-HT_2C_R antagonist SB242084 (0.5 mg/kg) in a between-groups serial design and Experiment 2 used 5-HT_2C_R KO mice in a repeated measures design. Animals initially learned to discriminate between two egocentric turning directions, only one of which was food rewarded (denoted CS+, CS−), in a T- or Y-maze configuration. This was followed by three conditions; (1) Full reversal, where contingencies reversed; (2) Perseverance, where the previous CS+ became CS− and the previous CS− was replaced by a novel CS+; (3) Learned non-reward, where the previous CS− became CS+ and the previous CS+ was replaced by a novel CS-. SB242084 reduced perseverance, observed as a decrease in trials and incorrect responses to criterion, but increased learned non-reward, observed as an increase in trials to criterion. In contrast, 5-HT_2C_R KO mice showed increased perseverance. 5-HT_2C_R KO mice also showed retarded egocentric discrimination learning. Neither manipulation of 5-HT_2C_R function affected performance in the full reversal test. These results are unlikely to be accounted for by increased novelty attraction, as SB242084 failed to affect performance in an unrewarded novelty task. In conclusion, acute 5-HT_2C_R antagonism and constitutive loss of the 5-HT_2C_R have opposing effects on perseverance in egocentric reversal learning in mice. It is likely that this difference reflects the broader impact of 5HT_2C_R loss on the development and maintenance of cognitive function.

## Introduction

Purposeful goal-directed responses may require an organism to flexibly adapt to changing situational demands by overcoming previously learned associations. This form of learning includes the ability to adjust responding following altered stimulus reward contingencies and is often assessed by reversal learning tests. Schizophrenia is characterised by cognitive deficits that precede and outlast other symptoms and predict long-term outcome [1]. These cognitive deficits include impaired reversal learning [2] observed as behavioural perseveration with patients showing inappropriate repetitive responding following a task contingency shift. Such perseveration may be produced by diverse cognitive impairments although the term is often associated with a potential explanation in terms of inappropriate stability of previous stimulus-reward associations. Available neuroleptics’ failure to treat these deficits severely limits treatment and can contribute to a poor long-term outcome [3].

Altered serotonin (5-Hydroxytryptamine, or 5-HT) signalling has been linked to a range of anatomical and cognitive abnormalities in schizophrenic patients. Lower brain levels of 5-HT in schizophrenic patients correlate with severity of cognitive impairment [4], hypofrontality during attentional set-shifting [2], [5], and poor long-term social and clinical outcome [3], [6]. These serotonergic abnormalities may involve altered signalling at the 5-HT_2C_ receptor (5-HT_2C_R). Schizophrenic patients show aberrant 5-HT_2C_R binding in the prefrontal cortex (PFC) [4], [7], decreased 5-HT_2C_R mRNA in the PFC [8], and altered PFC 5-HT_2C_R pre-mRNA editing [9].

Serotonin and the 5-HT_2C_R are also implicated in reversal learning. Acute tryptophan depletion can impair probabilistic reversal learning in healthy human subjects [10] and PFC or orbitofrontal cortex (OFC) specific 5-HT depletion retards visual reversal learning in the marmoset [11]. Similarly, systemic 5-HT depletions retard bowl-digging [12], go/no-go [13] and instrumental probabilistic reversal learning [14] in the rat. Improved allocentric visuospatial reversal learning is also seen in rodents systemically treated with the 5-HT_2C_R antagonist SB242084 [15], [16] and in 5-HT_2C_R knock-out (KO) mice [16].

Altered reversal learning performance may be caused by changes in the ability to overcome prior associations of either or both positive and negative valence. A rewarded two-choice discrimination can be reduced to an excitatory conditioned stimulus (CS) – unconditioned stimulus (US) association, eliciting approach, and an inhibitory CS – ‘no US’ association, eliciting withdrawal. Following a contingency shift, the CS initially predicating the US becomes associated with ‘no US’, a process opposed by *perseverance.* Conversely, the CS initially predicating ‘no US’ now predicts the US, a process opposed by *learned non-reward* [17].

Although behavioural perseveration defines a range of behaviours related to the excessive maintenance of activities, including inappropriate responding in the context of reversal learning [18], [19], it does not define the valence of the association that is inappropriately maintained. The term *perseverance* is used here to specify excessive responding towards previously rewarded stimuli in a task that attempts to dissect the underlying cognitive components of behavioural flexibility.

**Figure 1:**
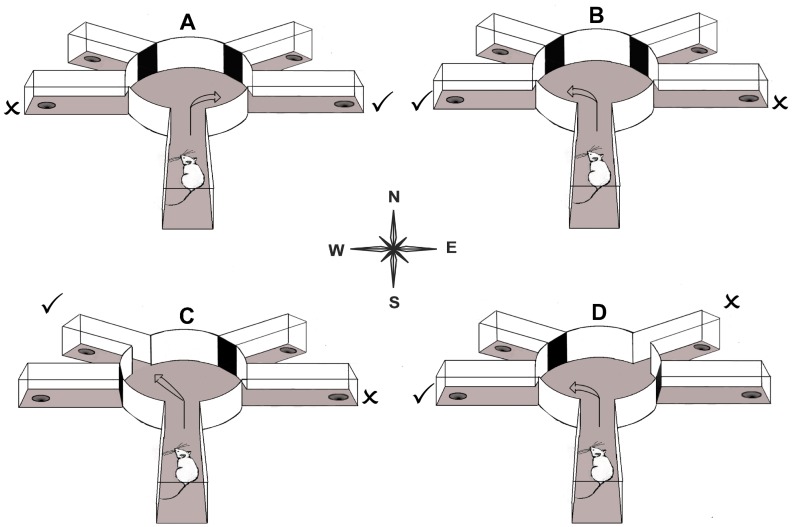
Diagram depicting the four types of discrimination. Example of the spatial discrimination (A) full reversal test (B), perseverance test (B) and learned non-reward test (C). Other maze arms not shown for clarity.

One approach to understanding the relative contributions of perseverance and learned non-reward has been to dissect tasks of cognitive flexibility into separate tests assessing these two processes by pairing a novel CS either with the previously correct CS or with the previously incorrect CS [11], [16], [17], [20], [21]. Here we investigate the role of the 5-HT_2C_R in reversal learning dissected into perseverance and learned non-reward using a spatial maze procedure. The task used egocentric discriminanda, no exteroceptive cues were provided to accurately guide responding. All testing took place in the dark using a radial-arm maze in multiple T- or Y-configurations in order to reduce the influence of any residual allocentric cues (Fig. 1).

Egocentric tasks have been used to assess the roles of dopamine (DA) and 5-HT signalling in reversal learning and discussed in relation to schizophrenia [22]–[24]. However, there have rarely been attempts to explore and replicate neuropharmacological manipulations across egocentric and allocentric spatial tasks of reversal learning. This becomes particularly pertinent considering that egocentric and allocentric spatial learning may require different underlying neural systems. For instance, rodent egocentric but not allocentric spatial learning has repeatedly been shown to be dependent upon the integrity of the dorsal striatum [25]–[28].

Experiment 1 assessed the effects of the 5-HT_2C_R antagonist SB242084 and Experiment 2 compared 5-HT_2C_R KO and wild type (WT) mice. The test conditions composed full reversal, or reversal in which either the previously incorrect or correct arm was replaced by a novel alternative, thus providing tests of perseverance and learned non-reward, respectively. A further experiment investigated the effect of a novel arm on unrewarded choice behaviour to demonstrate that the changed maze configuration is treated as a novel alternative by mice in this task and also to investigate potential effects of 5-HT_2C_R antagonism on responses to this novel alternative.

## Method

### Experiments 1 & 2– Egocentric Cognitive Flexibility

#### Animals

Experiment 1 used 72 C57BL/6J male mice (Charles River, UK) weighing a mean 24.9 g (SEM ±0.1) at the start of the experiment. Experiment 2 used 33 male mice bred at the University of Sussex (18 WTs; 15 KOs) weighing a mean 25.9 g (SEM ±0.4) at the start of the experiment. One week prior to food deprivation, animals were single housed in a controlled environment held at 21±2°C and 50±15% relative humidity with a 12∶12 h light-dark period (lights on at 07∶00 h). One week before commencing behavioural training, animals were food deprived to 85–90% of their *ad libitum* weight. During this week, animals were handled daily for 5–10 min after which 3–4 sucrose pellets were placed in each home-cage to reduce neophobia. On the last day of the week, animals in Experiment 1 received a sham saline injection (4 ml/kg) for habituation to the injection procedure. Animals were fed 2.5–3.0 g daily of standard laboratory chow (Special Diet Service Ltd, Witham, UK) 1 h after completion of behavioural training and testing. The experiments were licensed under the UK Animals (Scientific Procedures) Act 1986 (Project Licence 70/6654) following approval by the University of Sussex, Local Ethical Review Committee.

#### Apparatus

The experiments used an eight-arm radial maze made of clear Plexiglas elevated 55 cm above the floor. Each arm (33.5×5×8.3 cm) extended from a circular central platform (15.5 cm diameter). Access to the arms was controlled by inserting or removing clear Plexiglas inserts at the entrances to each arm. Black-painted vial bottle tops (80 mm diameter, 40 mm deep) figured as food-wells. The maze was enclosed by a featureless circular ‘tent’ of blackout material within which the maze could be rotated. A red light bulb and bullet-camera was located 63 cm above the central platform. The camera connected to a monitor and DVD recorder located in the corner of the room. Before a mouse was placed in the maze, this was always wiped with a sponge moistened with disinfectant to minimise intra-maze olfactory cues. The choice-behaviour of the animals was observed through the monitor, which was kept at minimal luminance to minimise visual cues.

#### Drug

SB242084 (6-chloro-5-methyl-1-[2(2methylpyridyl- 3-oxy)-pyrid-5-yl carbamoyl] indoline hydrochloride; Tocris, Bristol, UK) was initially dissolved in PEG400 (Sigma-Aldrich, Poole, UK) at 20% of the final required volume, which was then made up by 10% (w/v) hydroxypropyl-beta-cyclodextrin (Fluka, Poole, UK). The stock solution was aliquoted and frozen at −80°C in vials of quantities required for each test day. Each animal in Experiment 1 was dosed at 0.5 mg/kg subcutaneously (s.c.) in the nape of the neck at a volume of 4 ml/kg 30 min prior to behavioural testing.

#### Breeding and genotyping

The 5-HT_2C_R KO and WT animals used in Experiment 2 were of a C57BL/6J background generated as previously described [16]. The original progeny of 5-HT_2C_R KO mice used here were a gift from L. Tecott and produced as described by [29]. Wild-type male mice were crossed with females heterozygous for the X-linked 5-HT_2C_R mutation generating male WT and KO offspring. Genotyping was achieved using PCR on tissue samples from ear punches. The wild-type allele was detected using primers of the 5-HT_2C_R gene sequences flanking the Neo insertion: m5h2c (5′-AGTTGATGTTCATCTCAGGTGGC-3′) and 3N2 (5′-GGGTCCTATAGATCGAGGTACC-3′). The mutant allele was detected using primers complimentary to neomycin resistance gene (Neo) sequences: NeoD (5′-CACCTTGCTCCTGCCGAGAAA-3′) and NeoH (5′-AGAAGGCGATAGAAGGCGATG-3′). Breeding animals had been backcrossed for more than 20 generations and the individuals used here were 10–24 weeks old (age-matched for genotype) at the beginning of the experiment.

#### Behavioural procedure

Maze habituation. One week after the beginning of food deprivation, animals received four days of habituation to the apparatus configured as a cross-maze. The mouse was placed in the central area of the maze. On day one, five pellets were placed in each of the four arms (three along their lengths and two in the food-wells located at the end of each arm). This was gradually decreased over the four days. On the last day of maze habituation, only one pellet was located in each of the four food-wells. Each mouse was placed in the maze for a maximum of 3×12 min/day. Once all pellets were consumed or after 12 min had passed, the mouse was removed from the maze, the maze was re-baited, and the next habituation trial began. This procedure served to habituate the animals to the maze and to repeated handling. Between habituation trials, the mouse was placed in a holding cage with a heavy-absorbent paper on the floor in order to avoid the potential transfer of olfactory cues to the test apparatus. On the last day of maze habituation, animals consumed all of the pellets in the three habituation trials in a mean of 5 min.

Turn bias. The mouse turn-bias was determined after maze habituation and before discrimination learning. The maze was given a T- or Y-configuration with the start-arm being S (south), W (west) or E (east) across trials but never N (north). The maze configuration (Y-maze vs. T-maze) was counterbalanced across the different experimental groups. The mouse was placed in the start-arm and had the choice of turning left or right, with both arms baited in order to delay any association between response and reinforcement. The start-arm for each trial was predetermined in a pseudorandom order identical for each mouse. Each animal was given seven trials. A trial comprised one left and one right response. For example, if the mouse turned left, it was allowed to consume the pellet and thereafter returned to the start-arm to make a new choice. If choosing left once more, the mouse was immediately returned to the start-arm. The trial continued until the mouse had turned right. To calculate the mouse turn-bias, the first turn of each trial were summed, with the majority of responses determining the mouse turn-bias to left or right.

Spatial discrimination (Fig. 1A). Again, the start-arm was S, W or E across trials but never N. The start-arm for each trial was predetermined in a pseudorandom order identical for each mouse. Over every nine trials, each arm figured as the start-arm an equal number of times but never as a start-arm for more than two consecutive trials. The mouse had the choice of turning 90° (T-maze) or 45° (Y-maze) to the left and right. In spatial discrimination learning, the mice were always trained to turn against their own turn-bias. After approximately every 7th trial, the maze was rotated 90° to minimise extra-maze cues. After making a response, the mouse was removed from the maze and returned to the holding cage while the maze was set up for the next trial. The inter-trial interval was approximately 40 s. If a mouse made nine consecutive correct responses it was given a probe-trial. In the probe-trial, the use of an egocentric response strategy was pitted against the use of exteroceptive cues by using N as the start-arm. If successful, egocentric spatial discrimination was deemed completed and the animal was returned to its home-cage. If unsuccessful, a further five correct responses led to a new probe-trial. Each animal was given 25 trials/day. However, if the animal had completed ≥6 consecutive correct responses by the end of the 25th trial, it was given the chance to reach criterion. Nine consecutive correct responses followed by a correct probe trial was used as criterion in egocentric spatial discrimination learning as well as in all subsequent tests involving contingency shifts.

Full reversal test (Fig. 1B). Here the contingencies from the initial spatial discrimination were reversed. For example, an animal previously trained to turn right now had to turn left. Thus, the bait was moved to the opposite arm without any additional changes in the maze configuration.

Perseverance test (Fig. 1C). Here the previously correct arm remained opened while a novel arm replaced the previously incorrect arm. For example, a previously incorrect arm 90° to the left was replaced by a novel arm 45° to the left. Only the novel arm was baited. Hence, altered performance in this test condition must be due to a change in the association of reward, as the previously incorrect response alternative is no longer present. Thus the only acquired association that could influence choice behaviour in this test condition was the previous CS+.

Learned non-reward test (Fig. 1D). Here the previously incorrect arm remained opened while a novel arm replaced the previously correct arm. For example, a previously correct arm 90° to the right was replaced by a novel arm 45° to the right. Only the previously incorrect arm was baited. Hence, altered performance in this test condition must be due to a change in the association of learned non-reward, as the previously correct response alternative no longer is present. Thus the only acquired association that could influence choice behaviour in this test condition was the previous CS−.

### Experiment 3– Maze Novelty Recognition

#### Animals

Experiment 3 used 28 single housed WT C57BL/6J (Charles River, UK) male mice weighing a mean 26.2 g (SEM ±0.1) at the start of the experiment. The mice had *ad libitum* access to food and water throughout the experiment and the maze was not baited with food pellets.

#### Behavioural procedure

The experiment used the same apparatus as Experiment 1 and 2. Animals were initially habituated to a T-maze or a Y-maze for 3×12 min/day for three days. After each 12 min habituation trial, the animal was removed from the maze and placed in the holding cage while the maze was wiped with a disinfectant. Animals received sham saline injections (4 ml/kg) on the last two days for habituation to the injection procedure. Testing took place on the fourth day over 2×15 min. In the first 15 min phase of the test, the maze was maintained in the same configuration as during habituation. In the second 15 min phase of the test, one of the previously open arms was closed while an arm 45° to the north or south was opened. The maze configuration (T-maze vs. Y-maze) and location of the novel arm (N vs. S) were counterbalanced across the experimental groups.

### Experimental Designs and Statistical Analysis

Experiment 1 assessed the effects of the 5-HT_2C_R antagonist SB242084 on reversal learning, perseverance and learned non-reward using a between-subjects design. After completing the spatial discrimination drug-free, animals were pair-matched for trials to criterion and randomly assigned to a drug and test condition. Animals subsequently completed one of a full reversal test, perseverance test, or learned non-reward test.

Experiment 2 assessed 5-HT_2C_R KO mice on discrimination learning, reversal learning, perseverance and learned non-reward using a within-subjects design. All animals completed an initial spatial discrimination followed by a full reversal test. This was followed by a learned non-reward test and a perseverance test. The order of the perseverance and the learned non-reward tests was counterbalanced across the two genotypes.

In both Experiments 1 and 2, animals were allowed a maximum of 10 days (i.e., 250 trials) to reach criterion within each test condition. Animals failing to reach criterion on a test were assigned a trial-score of 250 for that test and not tested further. The dependent variables collected from each test condition were probe-trials, trials, correct responses and incorrect responses to criterion. To analyse performances in the early and late phases of learning, trials to criterion were broken down into 10-trial bins. Incorrect responses made before achieving 50% correct responses in a 10-trial bin were coded as early errors. Incorrect responses made once the animal achieved ≤50% correct responses in a 10-trial bin were coded as late errors.

In Experiment 1, the data was analysed by 2 (drug) × 3 (test condition) between-subject ANOVAs. Significant interactions were followed-up by separate ANOVAs or LSD post-hoc comparisons. In Experiment 2, a number of predominantly KO animals failed to complete criterion across all tests within 250 trials. Genotype differences in percent achieving criterion within each test was initially investigated by analysing the distribution of failing or passing through chi-square distribution analysis. Trials, correct and incorrect responses to criterion for each test was then analysed through one-way between-subject ANOVAs with genotype as the independent variable. Behavioural analyses only included animals attempting a given test.

In Experiment 3, animals were dosed with vehicle or 0.5 mg/kg of SB242084 15 min before testing. Hence, the novel-arm was introduced 30 min after drug treatment, as in Experiment 1. The 2×15 min test phases (pre- and post-change) were recorded and analysed using JWatcher (version 1.0). The proportion of time spent in each arm and the proportion of arm entries made into each arm was scored before and after the 45° change in response arm location. An arm-entry was scored once an animal had moved far enough into the arm that its hind paws were beyond the location of the insert between the central platform and the arm.

## Results

### Experiment 1: Effect of SB242084 on Egocentric Reversal Learning

Four of the 72 animals were excluded from analysis. Three of these animals failed to respond in the spatial discrimination test, and the remaining one was excluded after becoming ill. An additional two animals failed to complete the full reversal test within 250 trials (one in each drug condition). Animals assigned to the two maze configurations, three test conditions, or two drug groups did not differ in performance in the spatial discrimination (Table 1). There were no significant main effects of drug, test condition, or drug × test condition interaction on probe-trials to criterion (M = 1.5±0.07).

**Table 1:** Mean trials and incorrect responses (± SEM) to criterion in spatial discrimination in the SB242084 and 5-HT_2C_R experiments.

	SB242084 experiment(drug free)			5-HT_2C_R KO experiment		
	Vehicle	SB242084	p	WT	5-HT_2C_R KO	p
Trials	57.0±6.2	60.3±8.2	ns	55.2±7.2	85.5±10.3	.019
Incorrect	21.9±3.2	21.8±4.1	ns	17.9±3.0	35.9±7.1	.019

**Figure 2:**
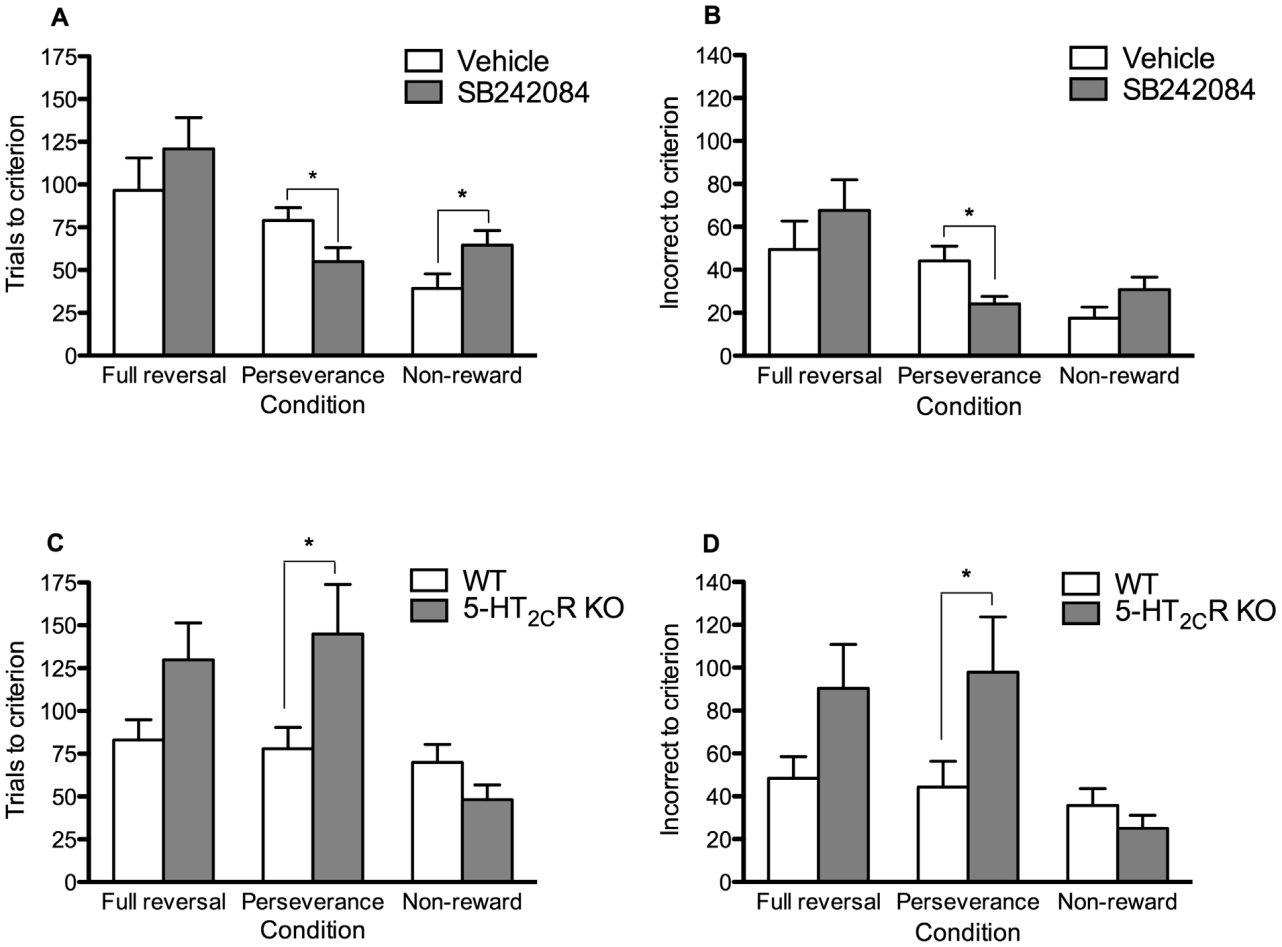
Performance in the full reversal, perseverance and learned non-reward tests in the SB242084 (a–b) and 5-HT_2C_R KO experiments (c–d). Asterisk denote differences at which p<.05. (a) Significant main effect of test (F_2,61_ = 13.10, p<.0001) and a near significant drug × test interactions (F_2, 61_ = 3.13, p = .051) on trials to criterion. SB242084 decreased trials to criterion in the perseverance test (F_1,20_ = 4.54, p = .046) and increased trials to criterion in the learned non-reward test (F_1,22_ = 4.44, p = .047). (b) Significant main effects of test condition (F_2, 61_ = 9.65, p<.0001) and drug × test interactions (F_2, 61_ = 3.46, p = .037) on incorrect responses to criterion. SB242084 decreased incorrect responses to criterion in the perseverance test (F_1,20_ = 5.96, p = .024). (c-d) Significant main effects of genotype on trials (F_1,26_ = 5.83, p = .023) and incorrect responses (F_1,26_ = 4.45, p<.045) to criterion in the perseverance test. Genotype had no significant effects on performance in the full reversal and learned non-reward tests.

SB242084 improved performance in the perseverance test, but retarded performance in the learned non-reward test (Fig. 2A, B). SB242084 failed to affect learning in the full reversal test. On trials to criterion (Fig. 2A), there was a significant main effect of test condition (F_2,61_ = 13.10, p<.0001). Animals required more trials to reach criterion in the full reversal test than in the perseverance (p<.001) and learned non-reward tests (p<.0001). There was also a near significant drug × test condition interaction (F_2, 61_ = 3.13, p = .051). Separate one-way ANOVAs showed that SB242084 decreased trials to criterion in the perseverance test (F_1,20_ = 4.54, p<.05), while increasing trials to criterion in the learned non-reward test (F_1,22_ = 4.44, p<.05). SB242084 had no effect on trials to criterion in the full reversal test (p = .20).

On incorrect trials to criterion (Fig. 2B), there was a significant main effect of test condition (F_2, 61_ = 9.65, p<.0001) and drug × test condition interaction (F_2, 61_ = 3.46, p<.05). Animals made more incorrect responses to criterion in the full reversal test than in the perseverance (p<.01) and learned non-reward tests (p<.0001). SB242084 decreased the number of incorrect responses made in the perseverance test (F_1, 20_ = 5.96, p<.05). Although the difference failed to reach significance, SB242084 treated animals made more incorrect responses in the learned non-reward test (F_1, 22_ = 2.97, p = ns). There were no significant effects of drug or drug × test condition interaction on early and late errors to criterion (p>.05; Table 2).

**Table 2:** Early and late errors to criterion in the three test-conditions of Experiment 1 and 2.

	Full reversal		Perseverance		Learned non-reward	
	Early errors	Late errors	Early errors	Late errors	Early errors	Late errors
**Experiment 1**						
Vehicle	34.8±9.9	14.7±4.5	33.3±6.4	10.8±4.0	14.9±4.2	2.7±1.5
SB242084	56.6±15.1	22.6±6	20.6±3.2	2.6±1	25.6±5.1	4.3±1.7
**Experiment 2**						
WT	31.4±7.6	17±4.3	31.3±12.4	13±3.2	26.3±7.7	9.4±1.7
5-HT_2C_R KO	67.3±18.3	23.1±6.2	71.4±26.2	26.5±9.7	19.5±5.9	5.5±1.9

*Experiment 1.* No significant effects of drug or drug × test condition interaction. Significant main effect of test condition on early (F_2,61_ = 5.37, p<.01) and late errors (F_2,61_ = 10.22, p<.0001). Animals made more early and late errors in the full reversal condition than the perseverance and learned non-reward conditions (p<.05). *Experiment 2.* No significant effects of genotype.

### Experiment 2: Effect of 5-HT_2c_R KO on Egocentric Reversal Learning

There were no effects of genotype on probe-trials to criterion (Grand mean = 1.4±0.06). No animals failed to complete the initial spatial discrimination or the learned non-reward test. However significantly more 5-HT_2C_R KO animals (N = 8) than WT animals (N = 2) failed to complete either the full reversal or perseverance tests (Table 3; χ^2^ = 7.53, p<.01).

**Table 3:** Proportion (%) of WT and 5-HT_2C_R KO mice reaching criterion in egocentric discrimination, full reversal, perseverance and learned non-reward tests.

	Egocentric discrimination	Full reversal	Perseverance	Learned non-reward
WT	100.0	94.4	94.1	100.0
5-HT_2C_R KO	100.0	73.3	63.6*	100.0

Significantly fewer 5-HT_2C_R KO animals than WT animals reached criterion in the perseverance condition (x^2^ = 4.2, p = .04).

5-HT_2C_R KO animals required more trials to criterion (F_1,31_ = 6.08, p<.05) and made more incorrect responses to criterion (F_1,31_ = 6.11, p<.05) in egocentric discrimination learning (Table 1). There was also a non-significant trend for 5-HT_2C_R KO animals to perform worse in the full reversal test by requiring more trials (F_1,31_ = 3.96, p = ns) and making more incorrect responses to criterion (Fig. 2C, D; F_1,31_ = 3.74, p = ns).

In the perseverance test, there was a significant effect of genotype on trials (F_1,26_ = 5.83, p<.05) and incorrect responses to criterion (F_1,26_ = 4.45, p<.05) with 5-HT_2C_R KO mice showing retarded learning relative to WTs. Although 5-HT_2C_R KO mice tended to perform better than WTs in the learned non-reward test, these differences were non-significant. There were no significant effects of genotype on early and late errors to criterion (p>.05; Table 2).

To further explore if the performance in the full reversal and perseverance tests could be accounted for by differences in initial discrimination learning, the data for these two test conditions were re-analysed using the initial spatial discrimination performance as a covariate. The near-significant effects of genotype on trials (Fig. 2C) and incorrect responses (Fig. 2D) to criterion in the full reversal test could be accounted for by retarded egocentric discrimination learning (trials, F_1,30_ = 0.64, p = .43; incorrect responses, F_1,30_ = 0.57, p = .46). However, the effect of genotype on performance in the perseverance test (Fig. 2C, D) remained statistically significant (trials, F_1,25_ = 7.65, p = .01; incorrect responses, F_1,25_ = 4.23, p = .05).

### Experiment 3: Effect of SB242084 on Unrewarded Choice Behaviour

Animals spent more time in the novel arm and made more arm-entries into the novel arm, and SB242084 had no effect on either of these measures (Table 4). There were no effects of maze-configuration or drug × maze-configuration interaction on entries into the novel or old arms. There were significant effects of phase (pre- and post-change) on proportion of time (F_1,26_ = 12.27, p<.01) and proportion of arm-entries made into the novel arm (F_1,26_ = 34.78, p<.0001). There were no effects of drug (p ≥.16) or drug × phase interactions (p ≥.19) on proportion of time spent in the novel arm or proportion of arm entries made into the novel arm.

**Table 4:** Proportion of time and proportion of entries (± SEM) before a 45° shift (pre-shift) and after a 45° shift (post-shift) in vehicle and SB242084 treated animals.

	Proportion of time (%)			Proportion of entry counts (%)		
	*Pre-shift*	*Post-shift*	*Change*	*Pre-shift*	*Post-shift*	*Change*
Vehicle	30.0±2.8	45.6±4.2	+15.6	34.2±1.4	47.6±2.7	+13.3
SB242084	35.5±3.5	43.0±3.1	+7.4	33.7±1.4	42.04±2.0	+8.3
Total	32.8±2.3	44.3±2.6	+11.5**	34.0±1.0	44.8±1.7	+9.5**

** p<.01.

## Discussion

Here we have investigated the involvement of the 5-HT_2C_R on egocentric discrimination and reversal learning using a T/Y maze-based task. Separable effects on perseverance and learned non-reward were revealed when the reversal learning task was dissected into its constituent cognitive components. Acute pharmacological antagonism of 5-HT_2C_R function attenuated perseverative responding but impaired responding to previously non-rewarded choices and, perhaps as a consequence, there was no effect on the full reversal task (Fig. 2A, B). However, genetic inactivation of these receptors in 5-HT_2C_R KO mice had opposite effects to those of acute 5-HT_2C_R antagonism and these mice also showed impaired egocentric discrimination learning in the initial phase of the maze task (Table 1). When 5-HT_2C_R KO mice were subsequently challenged with contingency shifts, they showed a selective increase in perseverative responding. There was no significant effect of genetic inactivation of the 5-HT_2C_R on either the learned non-reward or the full reversal task (Fig. 2C, D).

Interestingly, these results contrast with those from visuospatial instrumental assays in both rats [15], [30] and mice [16] suggesting that egocentric and visuospatial assays may depend upon different underlying neural systems. The inconsistent effects of genetic inactivation and acute antagonism on perseverative responding may have a parallel in the finding that 5-HT_2C_R antagonism has relatively small effects on nigrostriatal dopamine systems compared to those on the mesolimbic dopamine system [31] whereas the effects on nigrostriatal dopamine system function in 5-HT_2C_R KO mice are very much greater [32].

Also, both 5-HT_2C_R KO and SB242084 failed to affect early and late errors to criterion. In full reversal learning, early errors are often assumed to reflect the stability of the CS-US association, or perseverance, while late errors are assumed to be a measure of general cognitive abilities related to attention and the acquisition of an alternative CS-US association [33]. However, analyses of early and late errors are fundamentally different from the experimental manipulation of reversal learning currently used. As previously correct as well as incorrect CSs are presented in both early and late phases of full reversal learning, both associations may influence choice behaviour in both phases of learning.

### Generalisation and Novelty in the Egocentric Reversal Learning Task

In some variants of the task used here, animals were challenged with choice of a previously experienced response-arm and a novel response-arm. Animals may have generalised between the 90° and 45° turns in the same direction that were used to generate novel alternatives, and such generalisation would result in the perseverance and learned non-reward tests resembling tests of full reversal learning. However the test condition-dependent effects of SB242084-treated and 5-HT_2C_R KO mice, as well as the significantly increased number of trials required and larger number of incorrect responses made in the full reversal test than the learned non-reward and perseverance tests of the SB242084 experiment, suggest that animals perceived a 45° shift in arm location as novel. In Experiment 3, which relied on measures of unconditioned exploratory behaviour, a 45° shift in arm location led to significant increases in time spent and entries made into that arm, also suggesting it was recognised as novel.

Treatment-dependent changes in response to novelty may also affect performance in this task. The novel response arm is correct in the perseverance test but incorrect in the learned non-reward test. Increased novelty attraction would therefore facilitate learning in the perseverance test where the novel arm is correct, and retard learning in the learned non-reward test, where the novel arm is incorrect. Thus, one potential explanation for the pattern of results in the SB242084 experiment is that 5-HT_2C_R antagonism enhances responding for a novel choice in the maze. However, we are unaware of prior evidence for a role of the 5-HT_2C_R in novelty attraction or novelty recognition. SB242084 also failed to affect performance in the novelty recognition test (Experiment 3), suggesting the observed effects on cognitive flexibility (Experiment 1) instead are related to the ability to overcome previously learned contingencies of reward and non-reward.

Although the discrepant effects in visuospatial and egocentric tasks are most likely due to the tasks tapping different brain-regions and subpopulations of the 5-HT_2C_R, there are substantial differences in the two types of tasks. Additional to the use of different discriminanda, the current egocentric task involves perseverance to a greater extent than visuospatial reversal learning in the mouse [16]. The current protocol also involves less discrimination training than a visuospatial protocol in the rat [15], [30].

### Acute 5-HT_2C_R Antagonism and Egocentric Reversal Learning

Acute 5-HT_2C_R antagonism by SB242084 facilitated the ability to overcome perseverance, observed as a decrease in trials and incorrect responses to criterion. SB242084 also caused a concurrent impairment in the ability to overcome learned non-reward by increasing the number of trials to criterion. These opposing effects appear to have summated in the full reversal task, leading to no overall effect. It is very likely that these effects reflect 5-HT_2C_R blockade, rather than effects on another receptor mechanism. SB242084, especially at the relatively low dose used here, is highly selective for the 5-HT_2C_R, acting as a full antagonist or inverse agonist [34].

The observed effect of SB242084 in the perseverance test is in agreement with previous studies indicating that acute 5-HT_2C_R antagonism attenuates perseverative responding [15], [30]. It has been suggested that the SB242084-induced facilitation of operant lever reversal learning in the rat is related to decreased perseverance, as systemic or OFC-specific infusions of SB242084 can decrease repetitive responding towards the previous CS+ [30] or incorrect responses made early in reversal when responding is biased towards the previous CS+ [15]. In the learned non-reward test SB242084 impaired performance, seen as an increase in trials to criterion, in contrast to the effect seen in the perseverance test. This effect differs from the facilitating effects of SB242084 on learned non-reward in an instrumental analogue of the current protocol [16], indicating that egocentric and visuospatial reversal learning may involve different neural mechanisms.

Although little is known about the pharmacology of learned non-reward, work has been done in the closely related paradigm of latent inhibition, which like learned non-reward, could be thought of as the persistence of non-reinforced associations. In this paradigm, SSRIs and atypical antipsychotics can both elevate and attenuate latent inhibition depending on the number of pre-exposures and strength of the non-reinforced association [35], [36]. Interestingly, these two classes of compound do have 5-HT_2C_R antagonism as a common pharmacological property in addition to their other quite disparate effects [37], [38].

The effects of 5-HT_2C_R antagonism on visuospatial reversal learning have previously been discussed in relation to altered 5-HT and dopamine signalling [15], [39]. The 5-HT_2C_R receptor tonically inhibits dopamine (and noradrenaline) signalling in the PFC [40] and dorsal [32] and ventral striatum [41], However, this same group of studies show that the effects of 5-HT_2C_R antagonists on tonic serotonin signalling is much less evident. The implication may be that it is phasic release of serotonin that is responsible for the effects of these antagonists in the behavioural context of reversal learning [42].

### Genetic Inactivation of the 5-HT_2C_R and Egocentric Reversal Learning

5-HT_2C_R KO mice showed impaired egocentric spatial discrimination learning and, contrary to the effect of SB242084, they showed selective deficits in the subsequent perseverance test, observed as increased attrition rates, trials to criterion and incorrect responses to criterion, that could not be accounted for by the initial learning deficit. A recent study also reported opposing effects of SB242084 and constitutive loss of the 5-HT_2C_R in the 5-choice serial reaction time task [43].

Targeted mutations causing constitutive loss of specific components in 5-HT systems often cause adaptations additional to the mutation, leading to behavioural effects which differ from those of acute pharmacological blockade [44]. For example, the 5-HT_2C_R KO mutant show markedly elevated levels of dialysate DA in the dorsal striatum [32] while pharmacological inactivation is without effect on DA levels in this area [31], [45], [46]. Importantly, it has been speculated that perseverative responding can be produced by dorsal striatal DA elevations [47]. In a probabilistic reversal task, dopamine-agonist treated Parkinson patients show impaired performance compared to unmedicated patients [48] and increased dopamine activity at the D_2_R and D_3_R in the caudate nucleus, observed as an increase in methylphenidate induced [11C]-raclopride displacement in human volunteers, correlates negatively with reversal performance [49]. The selective increase in perseverative responding following genetic but not pharmacological inactivation could therefore be explained by the selective increase in dorsal striatal DA levels in the 5-HT_2C_R KO mouse.

Moreover, rodent egocentric spatial learning has repeatedly been shown to selectively depend upon the integrity of the dorsal striatum. For example, lesioning or local inactivation of the dorsal striatum impairs egocentric spatial but not allocentric visuospatial discrimination learning [25], [50] and working memory [26], [28] and dorsal striatal inactivation also impairs egocentric reversal learning [51]. Thus, the discrepant effects of 5-HT_2C_R inactivation across visuospatial [15], [16] and egocentric tasks could be explained by a greater involvement of the dorsal striatum in egocentric relative to allocentric spatial learning.

Alternatively, it is possible that both the impaired discrimination learning and perseverative responding seen in 5-HT_2C_R KO mice could be explained by altered functioning within the hippocampus. Aberrant spatial learning has previously been observed in 5-HT_2C_R KO mice using a water maze task [52]. Within the perforant path of the dentate gyrus, LTP-formation is suppressed both in the 5-HT_2C_R KO mouse [52] and by intraventricular 5,7-dihydroxytryptamine induced 5-HT depletions [53]. Since LTP-formation within the perforant path of the dentate gyrus correlate with spatial learning in the water maze [54] and blocking LTP-formation in the medial perforant path retards water maze performance [55], the observed retardation of discrimination learning could be related to the suppressed hippocampal LTP. However, there is no direct evidence to confirm that acute modulation of hippocampal 5-HT_2C_R function modulates either egocentric learning or the regulation of hippocampal LTP.

It should be recognised that the dichotomy that we have used between ‘visuospatial’ and ‘egocentric’ tasks may not fully reflect the differences between the types of task employed in rodent studies; particular task differences are also likely to be important. Specifically, impaired two-choice operant reversal learning has been observed in the 5-HT_2C_R KO mouse [43]. The reversal task described by Pennanen et al [43] is based on that described by Boulougouris et al [15], as is the one used in our earlier ‘visuospatial’ study [16]. However animals had to initiate individual trials in the two earlier studies by nosepoking into the magazine [15], [16], whereas trials were automatically initiated after a very short ITI in the recent report [43]. This is likely to have led to different behavioural strategies being used to ‘solve’ the task which themselves may be differentially sensitive to serotonergic manipulations. It may be that the perseverative impairments of 5-HT_2C_R KO mice in some task variants, including that used here and the one described in [43] are related to elevated dopamine dysregulation in the dorsal striatum or elsewhere.

### Concluding Remarks

Taken together the present results, in conjunction with previous studies, suggest that acute 5-HT_2C_R antagonism is likely to enhance reversal learning in visuospatial assays by acting on receptors located within the OFC. However there is likely to be more significant involvement of other areas, including the hippocampus, in the effects of such antagonists on reversal learning when animals perform an egocentric spatial task. Constitutive loss of the 5-HT_2C_R has more substantial effects on performance in the present egocentric spatial task which are likely to involve disturbance of function in additional brain areas, including the hippocampus and dorsal striatum.

Notably, behavioural perseveration may be produced by underlying cognitive deficits of perseverance and learned non-reward and influenced by other factors such as motor impulsivity. The wide range of tasks used to assess reversal learning is likely to pose very different demands and involve these processes to different extents and hence heterogeneity in results is to be anticipated.

The present findings may have relevance to the pathology and treatment of the cognitive deficits of schizophrenia, as the cognitive inflexibility deficits of the disease can be produced by specific deficits in perseverance [21]. Similar perseverative deficits were observed in 5-HT_2C_R KO mice, suggesting that a constitutive loss of the 5-HT_2C_R may be relevant for understanding the cognitive inflexibility that is characteristic of schizophrenia. Moreover, SB242084 facilitated the ability to overcome perseverative responding, while causing a concurrent increase in learned non-reward. As schizophrenia has been associated with both increased perseverance [21] and attenuated latent inhibition and learned irrelevance [56], [57], a tentative suggestion would be that the 5-HT_2C_R might be a pharmacologically relevant target for opposing both impairments.

In conclusion, the current results show that the 5-HT_2C_R modulates perseverative responding in an egocentric reversal learning procedure. The pattern of results indicates that serotonergic modulation of visuospatial and egocentric reversal tasks depends on different underlying neural systems and that constitutive loss of 5-HT_2C_ receptors leads to impaired acquisition of egocentric discriminations. An important challenge for future studies will be to specify the nature of these differences in both the tasks and experimental manipulations. This will have particular relevance preclinical tests used to characterise novel pharmacological treatments of human psychopathology.
